# Subtle Presentations and Diagnostic Complexities of Hydroxychloroquine Retinopathy: A Case Series

**DOI:** 10.7759/cureus.83393

**Published:** 2025-05-03

**Authors:** Alka Alex, Sherin M John

**Affiliations:** 1 Ophthalmology, Azeezia Institute of Medical Sciences and Research, Kollam, IND; 2 Ophthalmology, SJ Eye Hospital and Research Institute, Karukachal, IND

**Keywords:** drug side effect, hydroxychloroquine retinopathy, multidisciplinary care, rheumatoid arthritis, screening guidelines, spectral-domain oct

## Abstract

Hydroxychloroquine (HCQ) is widely used in managing autoimmune disorders but carries a notable risk of retinal toxicity, particularly with prolonged therapy. Early detection remains critical yet challenging, given the subtlety of initial clinical and imaging findings. We report a case series of three female patients (aged 68, 49, and 48 years), all of whom were on long-term HCQ therapy for rheumatoid arthritis and inflammatory polyarthritis.

Early retinal toxicity indicators such as subtle ellipsoid zone disruption, perifoveal pigmentary alterations, and initial visual field defects were overlooked in all three patients, leading to delayed recognition of HCQ-induced retinopathy. Despite robust screening recommendations and guidelines and comprehensive diagnostic evaluations including spectral-domain optical coherence tomography and multifocal electroretinography, practical challenges like inconsistent follow-ups and uncertainty in clinical decision-making complicated timely intervention.

These cases underscore the necessity of vigilant, structured, multimodal screening for HCQ retinopathy, emphasizing early recognition and proactive management. Improved clinician awareness, strict adherence to screening guidelines, and robust interdisciplinary coordination between ophthalmologists and prescribing physicians are essential to prevent irreversible visual impairment associated with HCQ use.

## Introduction

Since the 1950s, chloroquine and hydroxychloroquine (HCQ) have been used as anti-inflammatory medicines in chronic autoimmune conditions such as systemic lupus erythematosus, rheumatoid arthritis, dermatomyositis, Sjögren’s syndrome, and similar inflammatory conditions [1,2]. Adverse reactions of HCQ include gastrointestinal upset, skin rash, headache, and ocular changes. Ophthalmic side effects of these drugs include keratopathy, lens opacities, involvement of the ciliary body, and retinopathy [2]. HCQ is known to cause parafoveal retinal toxicity when administered at high doses and for prolonged periods. This toxicity is often insidious, causing serious, irreversible visual deterioration if not detected early. Recent representative studies have reported a prevalence of HCQ retinopathy ranging from 7.5% to 20%, depending on the duration of use of HCQ [3].

Early detection through structured ophthalmic screening is crucial but can be challenging due to subtle and easily overlooked clinical signs, particularly in the early stages of retinopathy [1,4]. Here, we present a case series of three patients to illustrate the different presentations of HCQ-induced retinopathy, highlighting the clinical characteristics, the diagnostic complexity, and the practical challenges encountered in following the recommended screening protocols.

## Case presentation

Patient 1

A 68-year-old female patient (weighing 58 kg) presented with inflammatory polyarthritis managed with HCQ 200 mg twice daily for approximately seven years. She underwent initial screening for HCQ toxicity in 2017.

**Figure 1:**
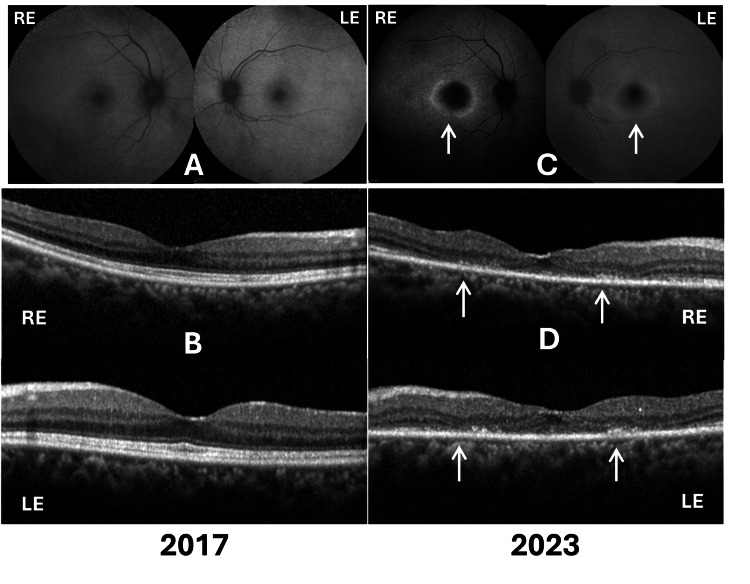
FAF and SD-OCT images from the right and left eyes from 2017 and 2023 Panel (A) shows normal FAF and panel (B) shows normal SD-OCT findings in 2017. Panel (C) demonstrates a hypoautofluorescent center surrounded by a hyperautofluorescent ring on FAF (white arrows), and panel (D) shows loss of ellipsoid zone, external limiting membrane, and outer nuclear layer on SD-OCT (white arrows) in 2023, consistent with advanced HCQ retinopathy. RE: right eye; LE: left eye; FAF: fundus autofluorescence; SD-OCT: spectral-domain optical coherence tomography; HCQ: hydroxychloroquine

At her initial evaluation in 2017, best corrected visual acuity (BCVA) was 6/9 in the right eye (RE) and 6/7.5 in the left eye (LE), with near vision recorded as N6 in both eyes. Slit-lamp biomicroscopy revealed an immature senile cataract in the RE. Fundus examination, fundus autofluorescence (FAF) (Figure 1A), spectral-domain optical coherence tomography (SD-OCT) (Figure 1B), and Humphrey visual field testing (HFA 24-2) were normal. Therefore, she was advised to continue HCQ with annual follow-up.

During a follow-up evaluation in 2019, the patient's visual acuity declined to 6/12 in the RE and 6/9 in the LE, maintaining N6 near vision bilaterally. Examination showed cataract progression in the RE. FAF imaging remained normal bilaterally. Subtle changes indicating ellipsoid zone (EZ) disruption with relative sparing of the subfoveal region on SD-OCT and peripheral scotoma on visual field testing were present but overlooked, and the patient continued HCQ therapy. Due to progressive vision reduction attributed to cataract, the patient underwent cataract surgery in the RE in February 2021. Postoperatively, visual acuity improved to 6/9 and N6 in the RE. In January 2022, the patient reported photopsia in the RE. The clinical fundus examination was normal; however, an HCQ screening was not performed during that time.

After a prolonged period without follow-up (approximately three years and eight months), she was referred by her rheumatologist to screen for HCQ-induced ocular changes in March 2023. A visual evaluation revealed BCVA of 6/9 in the RE and 6/12 in the LE, with near vision N6 bilaterally. Examination showed immature senile cataract in the LE, a posterior chamber intraocular lens (PCIOL) in the RE, and retinal pigment epithelium (RPE) changes in both maculae. FAF revealed central hypoautofluorescence surrounded by a hyperautofluorescent ring bilaterally, indicative of HCQ retinopathy (Figure 1C). SD-OCT demonstrated loss of EZ, external limiting membrane, and outer nuclear layer in the macula, with relative subfoveal sparing (Figure 1D). Wide-field OCT showed no abnormalities near the superior or inferior arcades. HFA 24-2 confirmed central scotomas bilaterally. Notably, earlier FAF imaging (2017, 2019) and wide-field OCT near vascular arcades remained normal throughout earlier evaluations.

After a diagnosis of HCQ-induced maculopathy, immediate cessation of HCQ was recommended, and a rheumatology review was arranged to adjust systemic medication accordingly. Counseling was provided regarding the irreversible nature of HCQ retinopathy, emphasizing the importance of regular ophthalmologic follow-up and adherence to screening guidelines. The patient was scheduled for a re-evaluation in six months to assess the progression or stabilization of retinal changes. During a follow-up visit after seven months, visual evaluation showed BCVA of 6/9 in the RE and 6/12 in the LE, with near vision N6 bilaterally, demonstrating that her vision parameters had not worsened since stopping HCQ.

Patient 2

A 49-year-old female patient (weighing 62 kg) presented in June 2023 with a history of rheumatoid arthritis managed with HCQ. She had been on HCQ 200 mg twice daily for seven years, later reduced to 200 mg daily for the past three years. She had been advised to stop HCQ at another center following suspicion of toxicity and presented for a second opinion.

**Figure 2:**
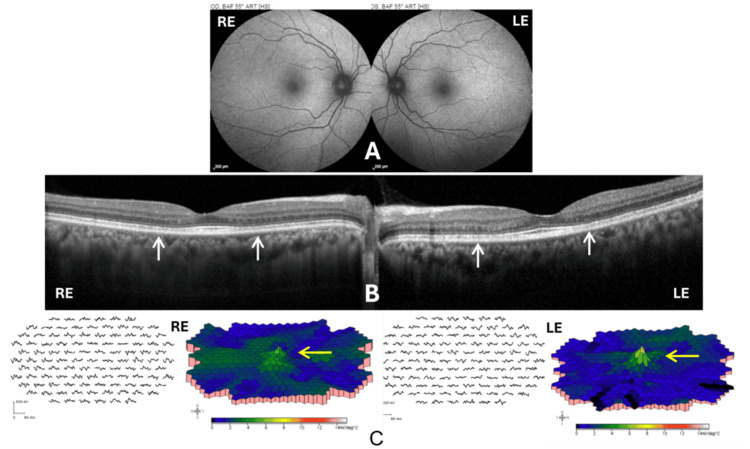
FAF, SD-OCT, and mfERG of both eyes Panel (A) shows normal FAF images. Panel (B) illustrates subtle bilateral ellipsoid zone disruptions with relative subfoveal sparing on OCT indicated by white arrows. Panel (C) shows mfERG results demonstrating reduced parafoveal and perifoveal responses indicated by yellow arrows, confirming early-stage hydroxychloroquine retinopathy. RE: right eye; LE: left eye; FAF: fundus autofluorescence; SD-OCT: spectral-domain optical coherence tomography; mfERG: multifocal electroretinography

Upon presentation, the patient's BCVA was 6/6 with near vision N6 bilaterally. Fundus examination demonstrated subtle pigmentary changes in the fovea bilaterally. Relevant negative findings included normal anterior segment examination without cataract or corneal abnormalities. FAF imaging was unremarkable, showing no signs of typical toxicity-associated hypoautofluorescent or hyperautofluorescent alterations (Figure 2A). SD-OCT demonstrated subtle EZ disruption bilaterally, with relative sparing of the subfoveal region (Figure 2B). Multifocal electroretinogram (mfERG) testing showed reduced parafoveal and perifoveal responses in both eyes, with diminished foveal peak amplitude in the RE and a mildly reduced peak in the LE (Figure 2C), consistent with early HCQ-induced maculopathy. Humphrey visual field testing was not documented, representing a potential omission of further corroborative findings.

Given the early yet definitive evidence of HCQ-related maculopathy, the patient was counseled regarding medication adjustment. It was recommended that the HCQ dosage be either reduced or switched to an alternative medication, in consultation with rheumatology. A six-month ophthalmologic follow-up was scheduled to monitor the progression or stabilization of retinal findings. Patient education was provided regarding potential visual risks, emphasizing adherence to ophthalmic screening guidelines and maintaining close communication with her rheumatologist. However, the patient has not presented herself for a review in our hospital yet.

Patient 3

A 48-year-old female patient (weighing 61 kg) with a six-year history of rheumatoid arthritis on HCQ presented for ophthalmic evaluation and HCQ toxicity screening. During this period, she was maintained at a dose of 200 mg twice daily.

**Figure 3:**
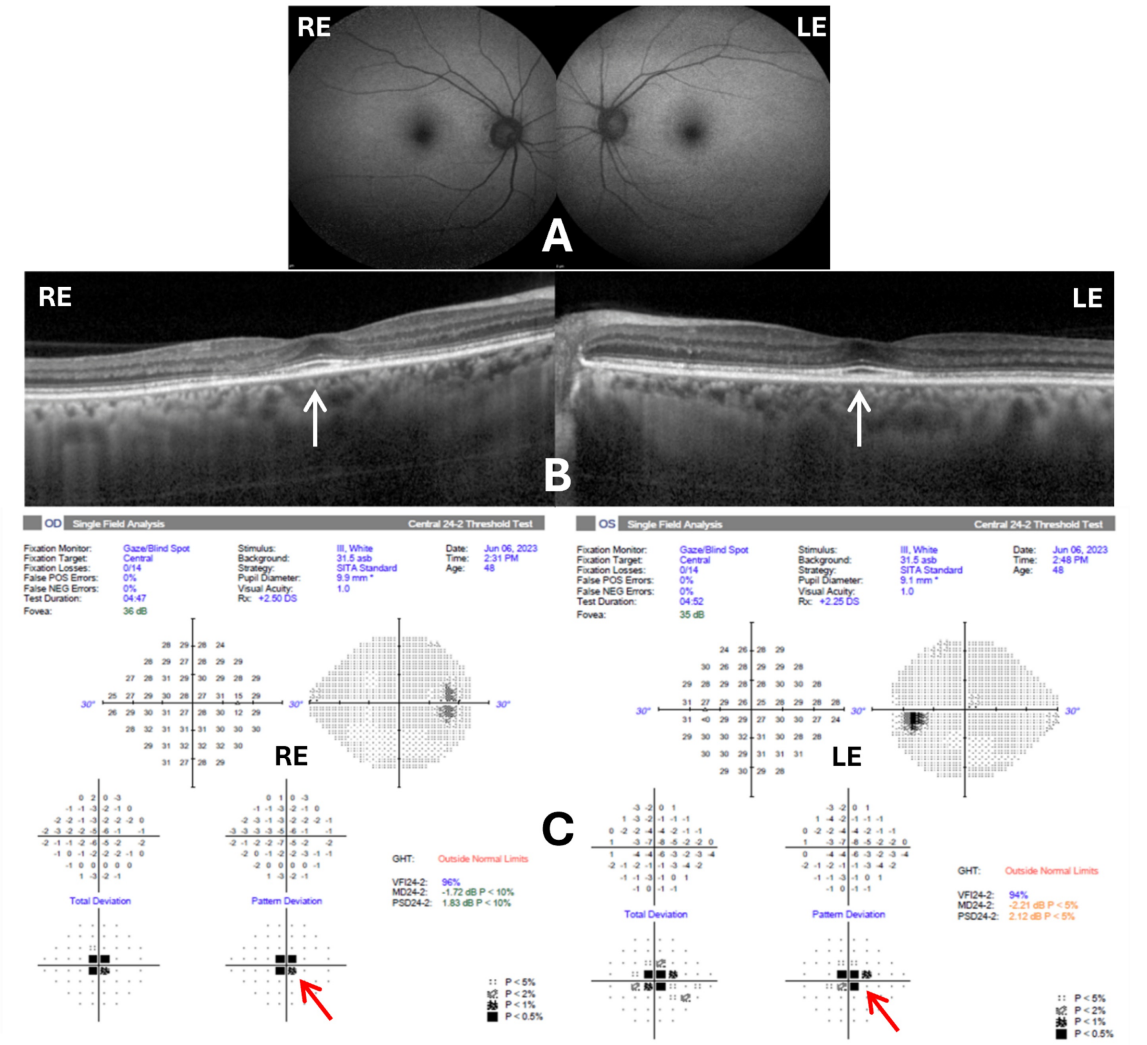
FAF, SD-OCT, and HFA 24-2 images for both eyes Panel (A) demonstrates normal FAF. Panel (B) shows focal ellipsoid zone disruptions, outer nuclear layer thinning nasal to the fovea, and an early “flying saucer” appearance on OCT (white arrows), characteristic of hydroxychloroquine-induced maculopathy. Panel (C) highlights central scotomas on visual field testing (red arrows), more significant in the RE. RE: right eye; LE: left eye; FAF: fundus autofluorescence; SD-OCT: spectral-domain optical coherence tomography; HFA 24-2: Humphrey visual field

Her BCVA at presentation was 6/6 bilaterally, with near vision N6. Anterior segment examination was normal bilaterally, with no evidence of cataract or corneal deposits. Fundus examination revealed RPE alterations at the fovea in both eyes. FAF imaging showed no abnormalities or characteristic HCQ toxicity patterns (hypo- or hyperautofluorescent changes were absent) (Figure 3A).

SD-OCT demonstrated focal EZ disruption with outer nuclear layer thinning nasal to the fovea in both eyes. The RE exhibited a notable early "flying saucer" appearance indicative of HCQ-induced toxicity. Both eyes showed relative subfoveal preservation (Figure 3B). HFA 24-2 confirmed a central scotoma more pronounced in the RE compared to the LE (Figure 3C). Relevant negative findings included normal FAF patterns, indicating early structural retinal changes without significant RPE loss.

Despite clear OCT and visual field evidence of HCQ maculopathy, the patient was advised to continue HCQ therapy but with close ophthalmologic monitoring due to her systemic disease activity and the potential challenges in medication adjustment. Dose modification or cessation was not recommended at this time, and this clinical decision-making required a careful re-evaluation. The patient was scheduled for a follow-up after six months for reassessment with repeat OCT and visual field testing. She was educated about early signs of potential visual deterioration and advised on strict adherence to ophthalmologic screening to closely monitor possible progression. However, the patient has not presented herself for a review in our hospital yet.

Table 1 provides a summary of these three cases.

**Table 1:** Patient demographics and summary of clinical findings HCQ: hydroxychloroquine; BCVA: best corrected visual acuity; OCT: optical coherence tomography; RE: right eye; LE: left eye; BE: both eyes; EZ: ellipsoid zone; ELM: external limiting membrane; RPE: retinal pigment epithelium; mfERG: multifocal electroretinogram

Patient	Age/gender/weight	Duration on HCQ	HCQ dose	Initial BCVA	Final BCVA	Key OCT findings	Visual field findings
Patient 1	68/F/58 kg	7 years	200 mg twice daily	RE: 6/9, LE: 6/7.5, N6 BE	RE: 6/9, LE: 6/12, N6 BE	EZ & ELM loss, macular RPE changes bilaterally	Central scotoma (bilateral)
								Patient 2	49/F/62 kg	10 years	200 mg twice daily for 7 years, then 200 mg once daily for 3 years	6/6 BE, N6 BE	6/6 BE, N6 BE	Subtle EZ disruption bilaterally, relative subfoveal sparing	Reduced parafoveal & perifoveal mfERG response
																Patient 3	48/F/61 kg	6 years	200 mg twice daily	6/6 BE, N6 BE	6/6 BE, N6 BE	EZ disruption nasal to fovea, early "flying saucer" appearance	Central scotoma (right > left)

## Discussion

HCQ is a widely prescribed medicine commonly used to treat autoimmune diseases such as rheumatoid arthritis and systemic lupus erythematosus because of its anti-inflammatory and immunomodulatory properties [1,2]. Although generally considered safe at the recommended doses, prolonged use of HCQ is associated with a well-documented risk of ocular toxicity, especially retinopathy, which may lead to severe and irreversible vision loss if not promptly detected [5].

HCQ retinopathy consists mainly of damage to the photoreceptors and the RPE, which usually manifests first as subtle parafoveal or pericentral changes [5,6]. As visual symptoms occur late in the course of the disease, early detection requires ophthalmologic examination using sensitive imaging techniques such as SD-OCT, FAF, automated visual fields (AVF), and mfERG [1,4,7-9]. Our series highlights the critical aspects of HCQ retinopathy in the context of evolving screening guidelines and real-world challenges.

Recent literature and guideline updates reinforce the importance of early, risk-based monitoring to detect toxicity before irreversible retinal damage occurs [1,4]. In 2016, the American Academy of Ophthalmology (AAO) updated its recommendation on screening, taking into account new data on prevalence, risk factors, and improved diagnostic tools [1]. Building on similar evidence, the Royal College of Ophthalmologists (RCOphth) issued comprehensive guidelines for the UK in 2018 and another review at the end of 2020, which refined the approach to monitoring for HCQ and chloroquine retinopathy [4]. It is noteworthy that the RCOphth 2020 guidelines do not support routine baseline screening of new HCQ and chloroquine users, due to evidence that very few patients stop treatment because of a baseline finding and many stop within the first five years for other reasons. Instead, patients with low risk factors are advised to start annual eye screening after five years of treatment, while for those with higher risk factors, screening should be started annually after one year of treatment [4].

Risk stratification remains key, with important risk factors including excessive daily dose (>5 mg kg daily), long duration of treatment (>5 years), renal impairment, concomitant use of tamoxifen, and pre-existing retinal or macular disease [1,5,6]. Ethnicity also has a significant impact on the presentation of HCQ retinopathy; in particular, Asian patients often present with extramacular (pericentral) involvement, requiring broader screening approaches [1,6]. While the presence of some ABCA4 gene polymorphisms may be protective, polymorphisms in cytochrome P450 gene might influence blood concentration and predispose patients to develop HCQ retinopathy. Recent exome sequencing and genome-wide association analyses revealed that RP1L1, RPGR, RPE65, and CCDC66 are among the candidate susceptibility genes linked to HCQ retinopathy [10].

Screening modalities emphasized by guidelines include SD-OCT and AVF. Each modality has limitations: subtle OCT changes can be overlooked, and visual field results can be variable or unreliable [1]. Hence, a multimodal approach combining these tests is advised for ambiguous findings [1,4].

Variable compliance with screening guidelines remains a challenge. Studies repeatedly show suboptimal compliance, reflecting gaps in communication and coordination between ophthalmologists and prescribing physicians [11]. Our cases illustrate how inconsistent monitoring can lead to delays in detection and preventable visual impairment. In addition to the emphasis on the guidelines, it is important to highlight the practical difficulties that clinicians face in applying them in their daily practice. Despite clear evidence and recommendations, variability in clinical practice regarding HCQ monitoring persists, and many ophthalmologists continue to report challenges in adhering to structured screening protocols [12,13]. This issue underscores the critical need for improved patient and clinician education, robust interventions in the healthcare system, and better interdepartmental coordination between ophthalmologists and prescribers, such as rheumatologists and dermatologists.

Recent advances in retinal imaging technology offer increased sensitivity and specificity for the early detection of retinopathy. Innovations such as OCT angiography (OCTA), adaptive optics (AO), and hyperspectral imaging offer a detailed view of the microstructure and function of the retina and may allow the earlier detection of HCQ toxicity in comparison with conventional methods [7-9,14]. When it came to disease-related changes, AI-based models, especially those that use full mfERG traces, showed superior predictive power than linear models. Although the dataset's imbalance may restrict some applications, this suggests significant potential to improve diagnostic capabilities [15]. However, incorporating these advanced technologies into routine screening requires further validation studies and cost-benefit analyses.

Ethnic-specific screening recommendations are evolving as clinicians gain a better understanding of genetic and environmental factors influencing drug metabolism and retinal susceptibility. Tailored screening strategies based on ethnicity could optimize the allocation of resources and increase early detection rates, especially in view of the evidence that Asian patients are more susceptible to pericentral retinopathy than Western patients [11]. Ongoing research into genetic predictors of HCQ retinopathy may potentially offer personalized screening and management plans in the future [10].

Improved multidisciplinary approaches to care are essential to reduce diagnostic delays and improve patient outcomes. Initiatives such as electronic medical record alerts, regular clinical audits, and integrated collaborative ophthalmology-rheumatology clinics have shown considerable potential to improve adherence to screening and early detection [6].

## Conclusions

A comprehensive understanding and application of the evolving guidelines on HCQ retinopathy, the integration of advanced imaging technologies, and enhanced collaborative clinical practice are essential to improve the outcome of patients and prevent irreversible vision loss. A proactive and informed clinical approach guided by evolving evidence-based practices is essential for the effective management of HCQ-induced retinopathy in the current ophthalmic practice.
